# Construction of a Macrophage Infiltration Regulatory Network and Related Prognostic Model of High-Grade Serous Ovarian Cancer

**DOI:** 10.1155/2021/1331031

**Published:** 2021-11-24

**Authors:** Hua Chang, Yuyan Zhu, Jiahui Zheng, Lian Chen, Jiaxing Lin, Jihang Yao

**Affiliations:** ^1^Department of Gynaecology, The First Hospital of China Medical University, Shenyang, China; ^2^Department of Urology, The First Hospital of China Medical University, Shenyang, China; ^3^Department of Urology, People's Hospital of Peking University, Beijing, China

## Abstract

**Background:**

High-grade serous ovarian cancer (HGSOC) carries the highest mortality in the gynecological cancers; however, therapeutic outcomes have not significantly improved in recent decades. Macrophages play an essential role in the occurrence and development of ovarian cancer, so the mechanisms of macrophage infiltration should be elucidated.

**Method:**

We downloaded transcriptome data of ovarian cancers from the Gene Expression Omnibus and The Cancer Genome Atlas. After rigorous screening, 1566 HGSOC were used for data analysis. CIBERSORT was used to estimate the level of macrophage infiltration and WGCNA was used to identify macrophage-related modules. We constructed a macrophage-related prognostic model using machine learning LASSO algorithm and verified it using multiple HGSOC cohorts.

**Results:**

In the GPL570-OV cohort, high infiltration level of M1 macrophages was associated with a good outcome, while high infiltration level of M2 macrophages was associated with poor outcomes. We used WGCNA to select genes correlated with macrophage infiltration. These genes were used to construct protein-protein interaction maps of macrophage infiltration. IFL44L, RSAD2, IFIT3, MX1, IFIH1, IFI44, and ISG15 were the hub genes in the network. We then constructed a macrophage-related prognostic model composed of CD38, ACE2, BATF2, HLA-DOB, and WARS. The model had the ability to predict the overall survival rate of HGSOC patients in GPL570-OV, GPL6480-OV, TCGA-OV, GSE50088, and GSE26712. In exploring the immune microenvironment, we found that CD4 memory T cells and activated mast cells showed that the degree of infiltration was higher in the high-risk group, while M1 macrophages were the opposite, and HLA molecules were overexpressed in the high-risk group.

**Conclusion:**

We constructed a macrophage infiltration-related protein interaction network that provides a basis for studying macrophages in HGSOC. Our macrophage-related prognostic model is robust and widely applicable. It predicts overall survival in HGSOC patients and may improve HGSOC treatment.

## 1. Introduction

Ovarian cancer is a highly malignant gynecological tumor usually found in the advanced stage. Global cancer data statistics in 2020 showed that there were 313959 new cases of ovarian cancer and 207252 new ovarian cancer deaths [1]. High-grade serous ovarian cancer (HGSOC) is the most common subtype of ovarian cancer, accounting for 75% of ovarian cancer and 70% of deaths [2]. HGSOC has a specific genetic susceptibility, and about 15–20% of HGSOC patients show BRCA1 or BRCA2 germline mutations [3]. HGSOC frequently acquires and loses DNA, making chromosomes in these cancers unstable and increasing the risk of acquired chemotherapy resistance [4]. Platinum chemotherapeutic drugs are the first-line treatment of HGSOC, and immune-related therapy is a new treatment modality [5]. However, the long-term survival from ovarian cancer has not significantly increased in the past three decades. Currently, prognostic factors of HGSOC include the FIGO stage, residual disease, BRCA1/2 germline mutations, and tumor-infiltrating lymphocyte score. However, these prognostic factors have great limitations, and the prediction effect is not ideal. Therefore, the construction of a molecular prognostic model of ovarian cancer is the focus of future research [5].

Macrophages play a complex and important role in tumor microenvironment. Resting macrophage are polarized into proinflammatory or anti-inflammatory forms [6]. M1 macrophages have properties of cytotoxicity, tumor inhibition, and immunostimulation functions, while M2 macrophages promote tumor growth and invasion [7]. Tumor-associated macrophages (TAM) are macrophages infiltrating around tumor cells, which are closely related to the malignant progression and clinical prognosis of ovarian cancer. In recent years, research on the targeting strategy of TAM has achieved great success. TAM targeting strategies include inhibition of macrophage recruitment, increase in M1 macrophages, and inhibition of the tumor-promoting activity of M2 macrophage [8]. Studies have also illustrated a relationship between the outcome of ovarian cancer and macrophages. The ratio of M1/M2 to M2/TAM was positively associated with overall survival and disease-free survival [9, 10]. The density of M2 macrophage in tumor samples was associated with decreased recurrence-free survival [11]. These studies suggest that macrophages or related markers are potential prognostic factors for ovarian cancer.

In this study, we calculated the level of macrophage infiltration and evaluated the potential of macrophages as prognostic markers. We constructed a gene coexpression network and identified the macrophage-related gene modules. We used the module genes to build a regulatory network related to macrophage infiltration. Then, a prognostic model related to macrophage infiltration was constructed using machine learning and verified several datasets. Finally, we explored the relationship between the model and immune factors and carried out enrichment analysis to determine the differences in signal pathways under different groups.

## 2. Materials and Methods

### 2.1. Data Download and Collation

**Figure 1:**
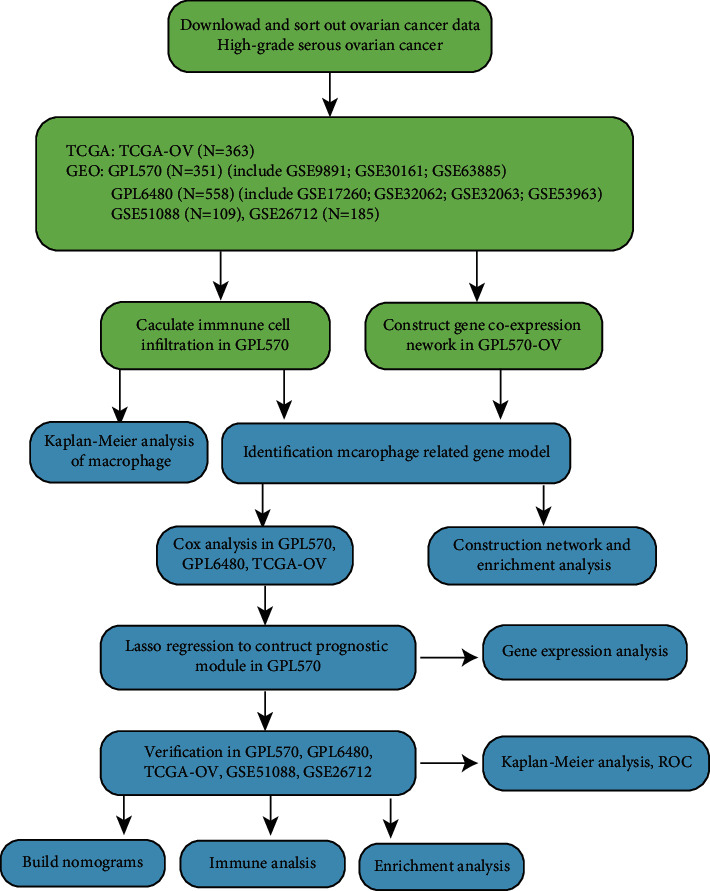
The flow chart describing our protocol.

We downloaded GSE9891 [12], GSE30161 [13], and GSE63885 [14] from Gene Expression Omnibus (GEO, https://www.ncbi.nlm.nih.gov/geo/). These data are based on the GPL570 platform. We downloaded GSE17260 [15], GSE32062 [16], GSE32063 [16], and GSE53963 [17] from GEO. These data are based on GPL570. We also downloaded GSE51088 [18], which is based on GPL7264, and GSE26712 [19], which is based on GPL96. Finally, the TCGA-OV cohort was downloaded from The Cancer Genome Atlas database (TCGA, https://portal.gdc.cancer.gov/). We collated these data and screened HGSOC samples with both transcriptome data and follow-up information. To reduce the prognostic changes caused by different treatments after surgery, we only selected samples that involved platinum-containing medication therapy. We then used the R package “sva” to merge the chip data and delete the batch effect from the same platform. Finally, there were 1566 samples remaining with GPL570 (*n* = 351), GPL6480 (*n* = 558), GSE51088 (*n* = 109), TCGA-OV (*n* = 363), and GSE26712 (185). The research process is illustrated in Figure 1.

### 2.2. CIBERSORT

CIBERSORT is an algorithm based on the principle of linear support vector regression, which uses immune cell subtype expression matrix for deconvolution [20]. LM22 in CIBERSORT is a signature gene expression matrix used to estimate the proportion of leukocytes in bulk RNA. Under the operation of the R package “cibersort” and LM22 matrix, we can calculate the composition proportion of 22 kinds of leukocytes of new samples.

### 2.3. Survival Analysis

Kaplan-Meier analysis is a univariate survival analysis. Through using the R packages “survival” and “survminer,” we identified the best cut-off value by the function “res.cut.” Receiver operating characteristic curves (ROC) were drawn according to a series of binary classifications. The area under the curve is the AUC value. When the AUC value is greater than 0.5, the result is statistically significant. Univariate Cox regressions were used to identify features related to survival, and multivariate Cox regressions were used to identify multiple features related to survival. Nomograms are multivariate regression analyses that integrate several predictive indicators and use graduated line segments to perform predictive analysis. Calibration curves are scatter plots of actual incidence and predicted incidence to evaluate logistic regression models. These analyses were performed by the R package “survival.”

### 2.4. Weighted Gene Correlation Network Analysis (WGCNA)

WGCNA is a method for analyzing gene expression patterns in multiple samples [21]. The calculation of WGCNA is based on the R package “WGCNA.” WGCNA was used to identify highly related gene sets and to construct coexpression networks of gene sets. First, the Pearson correlation between every two genes is calculated. Second, the most suitable *β* value is calculated to make the network satisfy the scale-free distribution; then the weighted correlation adjacency matrix is constructed by *β* value. Third, the topological overlap matrix is calculated to add some indirect interactions. Finally, the dynamic cut tree method is used to cluster genes to form gene modules. Genes clustered into the same module indicate that they may have similar functions. Pearson correlation analysis was performed between gene module and clinical trait data. Then we can explore the most relevant gene modules for specific clinical traits.

### 2.5. Construction of Protein-Protein Interaction Network

STRING data is a database for searching protein interaction networks (https://string-db.org/). Cytoscape is the network map visualization software, and we import the STRING results into the Cytoscape to draw protein-protein interaction network [22].

### 2.6. Enrichment Analysis

Metascape is a powerful gene annotation software package (https://metascape.org/) [23] used to recognize protein or gene function cognition. The database is updated monthly. Gene set enrichment analysis (GSEA) is an enrichment method used to study whether there is a statistically significant difference in the expression level of a given group of genes between the two biological states. Gene set variation analysis (GSVA) is a nonparametric analysis method used to calculate pathway enrichment.

### 2.7. Least Absolute Shrinkage and Selection Operator (LASSO)

LASSO is a linear regression method using L1 regularization. Through the operation of a penalty function for compressing partial regression coefficients, a more refined model is obtained. The process of building a model using LASSO is based on the R language, mainly using R packages “glmnet” and “survival.” First, the “glmnet” function was randomly simulated 1000 times to construct the model. Then the relationship between penalty coefficient lambda and gene coefficient was established. With the increase of lambda, some gene coefficients become zero, indicating that the gene is an invalid gene of the model. Then the 10x cross-validation is repeated 1000 times using the random simulation function “cv.glmnet.” When the deviation is minimum, the constructed model was the best, and then the corresponding lambda value was used to calculate the gene coefficient. Finally, we obtained the following prognostic model: risk score = ∑*n*_*i*_ (exp_*i*_·coef_*i*_) (where *n* is the number of genes, exp_*i*_ is the expression of the *i*th gene, and coef_*i*_ is the regression coefficient of the ith gene). We use the R package “survminer” to obtain the best cut-off value of the risk score and separate the cohort into high-risk and low-risk groups [22].

### 2.8. Gene Expression Analysis

GEPIA is an online website of gene expression based on TCGA database (http://gepia.cancer-pku.cn/) [24]. We used the TIMER website to query the expression of genes in pan-cancer (https://cistrome.shinyapps.io/timer/) [23]. The Human Protein Atlas is a database (https://www.proteinatlas.org/) that provides protein expression in human tissues. Cancer Cell Line Encyclopedia (CCLE, https://portals.broadinstitute.org/ccle/) is used to query gene expression, mutation, copy number, and methylation of thousands of cell lines from tissue origin [25]. We used those tools to query genes expression characteristics.

### 2.9. Single-Cell Data Analysis

TISCH (http://tisch.comp-genomics.org/) is an scRNA-seq database focused on the tumor microenvironment [26]. TISCH provides detailed cell type annotations at the single-cell level. CancerSEA (http://biocc.hrbmu.edu.cn/CancerSEA/) drew a map of 14 functional states by the scRNA-seq data. It allows users to query the potential pathways of specific genes in tumors [27].

### 2.10. Statistical Analysis

The statistical analysis used in this study is based on the environment of R language software (Rx64 3.5.1). Venn diagrams were drawn using online tools (http://bioinformatics.psb.ugent.be/webtools/Venn/). The risk score's distribution map was generated by the R package “pheatmap.” The box and correlation scatter charts were drawn by R packages “limma” and “ggplot2.”

## 3. Results

### 3.1. Calculating the Infiltration Level of 22 Kinds of Leukocytes in GPL570-OV

**Figure 2:**
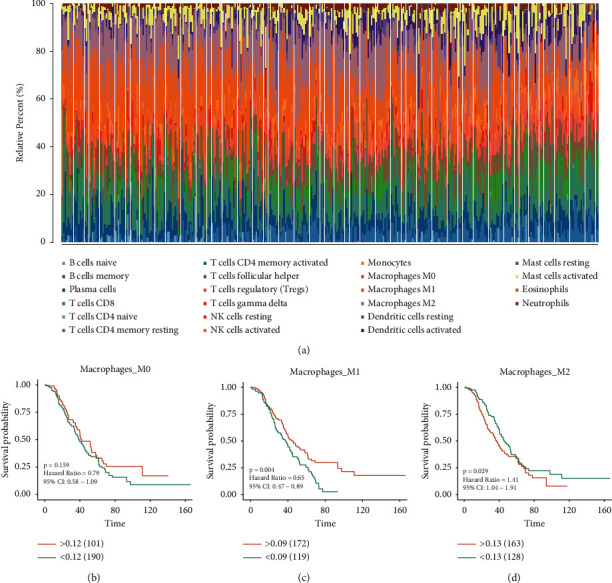
The proportion of leukocyte infiltration and survival analysis of macrophages. (a) The infiltration patterns of 22 leukocytes are shown in the bar graph. (b) Kaplan-Meier analysis of M0 macrophages. (c) M1 macrophages. (d) M2 macrophages. A *P* value less than 0.05 indicates statistical significance.

GPL570-OV had a total of 351 HGSOC samples, and the number of genes was 20486. We used CIBERSORT to calculate 22 leukocytes infiltration ratios in each sample (Figure 2(a)). We then extracted the infiltration levels of M0, M1, and M2 macrophages and used Kaplan-Meier analysis to determine whether the infiltration of the three kinds of macrophages affected the outcome. We found that outcomes in HGSOC with high infiltration level of M1 macrophages were better (Figure 2(c)), while that of HGSOC with high infiltration level of M2 macrophages was poor (Figure 2(d)). Although the result of M0 macrophages was not statistically significant, the outcome was better in the case of high infiltration (Figure 2(b)).

### 3.2. Construction of Coexpression Network Related to Macrophage Infiltration

**Figure 3:**
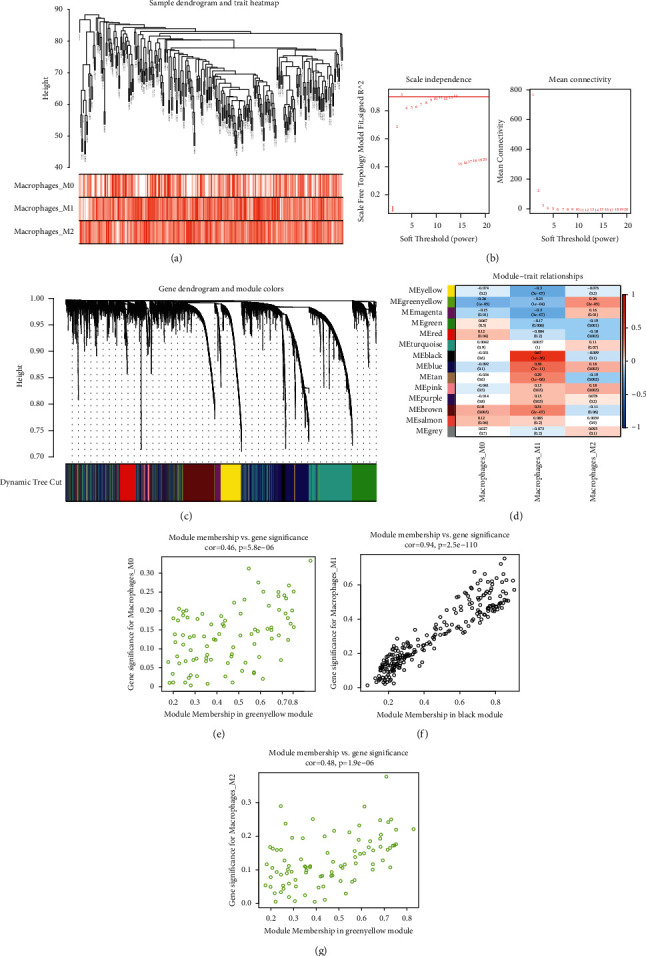
The gene weighted coexpression network. (a) Cluster analysis of samples. (b) The most appropriate soft threshold. (c) The dynamic cut tree is used for cluster analysis. (d) The correlations between gene modules and macrophages. (e) Gene significance scatters diagram of module membership and M0 macrophages of the green-yellow module. (f) Black module and M1 macrophages. (g) Green-yellow module and M2 macrophages.

In the expression network, genes with small gene expression variation represent noise; therefore, we calculated the coefficient of variation of each gene in the GPL570-OV cohort and took the coefficient of variation of the first 8000 genes according to their size to enter the subsequent analysis. First, we clustered the samples, eliminated the samples with high discrete degrees (Figure 3(a)), and then calculated the soft threshold *β*. We found that when *β* = 3, *R*^2^ was greater than 0.8, and the mean connectivity was as large as possible (Figure 3(b)). We use soft threshold to build scale-free network and then use dynamic tree cutting method to divide genes into different modules. We obtained 14 gene modules in this manner (Figure 3(c)). We calculated the Pearson correlation between the modules and macrophages (Figure 3(d)) and found that the black module had the highest correlation with M1 macrophage (*R* = 0.67). In contrast, the green-yellow module negatively correlated with M0 macrophage (*R* = -0.26) and positively correlated with M2 macrophage (*R* = 0.26). Figures 3(e)–3(g) are scatter diagrams of module membership and gene significance.

### 3.3. Construction of Macrophage Infiltration-Associated Protein Interaction Network

**Figure 4:**
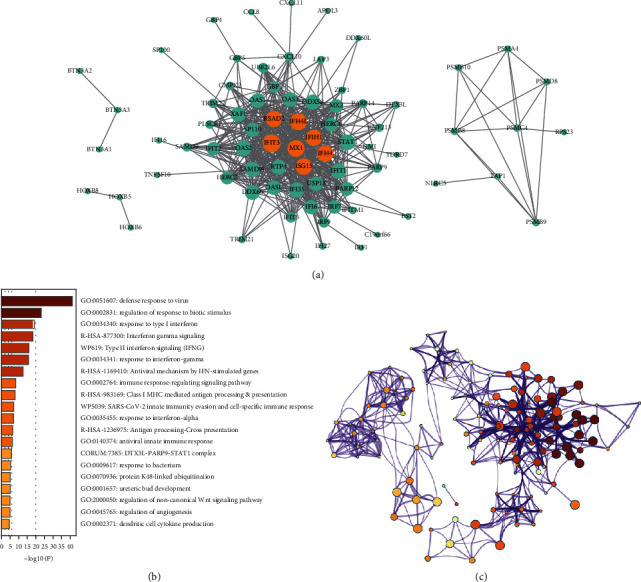
Analysis of gene module. (a) Protein-protein interaction network. (b) Metascape enrichment analysis. (c) Network interaction diagram of enriched items.

We took the genes in the green-yellow and black modules as macrophage-related genes to enter the following analysis. There were 89 genes in the green-yellow module and 234 genes in the black module. We input these 323 genes into the STRING database and built a gene interaction network. We found that IFL44L, RSAD2, IFIT3, MX1, IFIH1, IFI44, and ISG15 showed high connectivity and were at the center of the network (Figure 4(a)). After 323 genes were inputted into Metascape for enrichment analysis (Figures 4(b)–4(c)), we found that these genes were significantly enriched in immune-related pathways, such as “defense response to virus” and “regulation of response to biotic stimulus.”

### 3.4. Construction of a Macrophage-Related Prognostic Model

**Figure 5:**
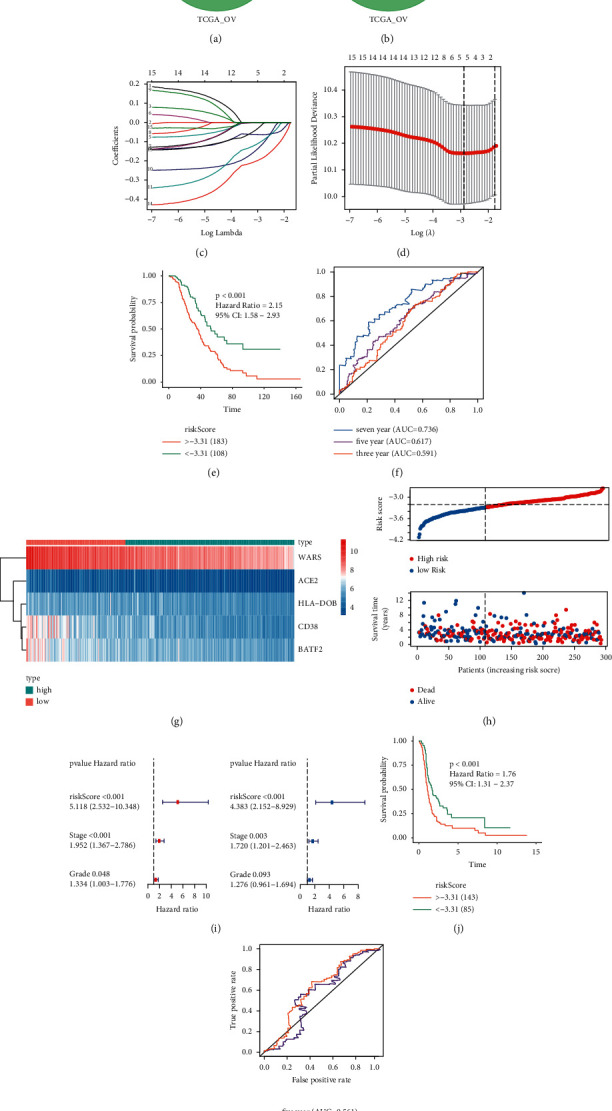
Construction of a prognostic model. (a) Venn map of protective genes. (b) Venn map of risk genes. (c) Fifteen gene coefficients and lambda curves. (d) 10x cross-validation. (e) Kaplan-Meier OS analyzing the risk score in GPL570-OV. (f) ROC of the OS for the risk score in GPL570-OV. (g) Expression heat map of five genes. (h) Distribution map of the risk score. (i) Univariate and multivariate Cox analysis. (j) Kaplan-Meier PFS analysis of the risk score in GPL570-OV. (k) ROC of the PFS for the risk score in GPL570-OV.

We performed univariate Cox analysis of 323 macrophage infiltration-related genes in GPL570-OV, GPL6480-OV, and TCGA-OV cohorts and selected the statistically significant results (Figures 5(a)–5(b)). Results showed 15 protective genes (HR < 1 and *P* < 0.05) and no risk genes (HR > 1 and *P* < 0.05) were obtained. We selected these 15 genes for LASSO analysis in the GPL570-OV cohort as the training set. With the increase of lambda value, the coefficient of some genes decreased to 0, suggesting that the contribution of these genes to the model is small and should be abandoned (Figure 5(c)). Then 10x cross-validation was carried out; when the number of genes was 5, the model reached the optimal solution (Figure 5(d) and Table 1). The formula of the model was as follows: risk score = CD38 *∗* (−0.063) + ACE2 *∗* (−0.121) + BATF2 *∗* (−0.100) + HLA-DOB *∗* (−0.017) + WARS *∗* (−0.019). Kaplan-Meier analysis shows that the model can carry out risk stratification of GPL570-OV (Figure 5(e), *P* < 0.001). The ROC curve showed that the AUC values of 3/5/7 years were all greater than 0.5, and the predictive ability of 7 years was the best (Figure 5(f)). We drew the expression heat map of five genes (Figure 5(g)) and the distribution map of risk scores (Figure 5(h)). Univariate and multivariate analysis showed that the model was predictive and independent of clinical stage and pathological grade (Figure 5(i)). Finally, we measured the ability of the risk score to predict progression-free survival (PFS) in GPL570-OV. The model maintains a good ability of hazard stratification (Figure 5(j), *P* < 0.05). The AUC values of three years and five years were greater than 0.5 (Figure 5(k)).

### 3.5. Analysis of the Expression Characteristics of Five Genes

We used the TIMER database to analyze the differential expressions of CD38, ACE2, BATF2, HLA-DOB, and WARS between various tumor tissues and normal tissues. The results show that the five genes had different expression characteristics in different tumors. For example, CD38, BATF2, HLA-DOB, and WARS were overexpressed in Head and Neck squamous cell cancer, while they were lower in Kidney Chromophobe (Supplementary Figure 1). We examined the differential expression value of five genes in 426 ovarian cancer tissues and 88 normal tissues in GEPIA. It was found that only the differential expression of WARS was statistically significant, and it was overexpressed in ovarian cancer. Although CD38, BATF2, and HLA-DOB have no statistical significance, they show an increasing trend of expression in ovarian cancer (Supplementary Figures 2A–2E). The mRNA level of CD38, ACE2, BATF2, and HLA-DOB is relatively low, with an average of 0–3, while the mRNA level of WARS is relatively high, with an average of more than 6. The results were consistent with the immunohistochemistry results (Supplementary Figures 2F–2J), and the protein level of WARS was relatively higher than the other four genes. WARS is mainly expressed in nuclear, while the other four genes are expressed in cytoplasmic/membranous. We also investigated the expression values of five genes in ovarian cancer cell lines (Supplementary Figure 2K). It was found that the expression of mRNA in ovarian cancer cell lines showed the same result, and the mRNA expression level of WARS was significantly higher than those of the other four genes.

### 3.6. Analysis of Model Gene Expression at the Single-Cell Level

In the CancerSEA database, we queried the related station of ACE2, BATF2, and WARS (Supplementary Figure 3A). We found a positive correlation between ACE2 and angiogenesis/hypoxia/metastasis/quiescence/inflammation pathway (cor > 0.3, *P* < 0.05) and a negative correlation between ACE2 and DNA-damage/DNA-repair/Invasion (cor < −0.3, *P* < 0.05). There was a negative correlation between BATF2 and the invasion/stemness pathway (cor < −0.3, *P* < 0.05) and there was a positive correlation between BATF2 and the quiescence pathway (cor > 0.3, *P* < 0.05). We extracted the GSE115007 dataset from the TISCH database and measured expression levels of CD38, BATF2, HLA-DOB, and WARS in immune cells. We found that expression levels of CD38 and BATF2 were low in immune cells (Supplementary Figures 3B-3C), while WARS was expressed in M2 macrophages, monocyte, plasma, cDC1, and cDC2 (Supplementary Figure 3D). HLA-DOB was significantly overexpressed in cDC1 and slightly expressed in cDC2 (Supplementary Figure 3E).

### 3.7. Verifying the Prognostic Ability of Macrophage-Related Prognostic Model in GPL6480-OV, TCGA-OV, and GSE50088

**Figure 6:**
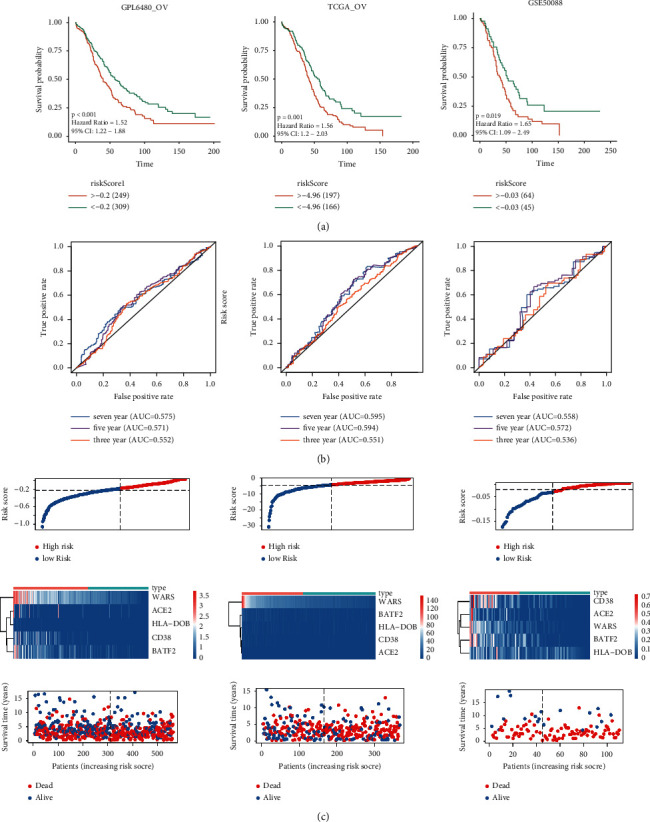
The prognostic ability of the model is verified in the verification set. (a). Kaplan-Meier analysis of risk scores performed in GPL6480-OV, TCGA-OV, and GSE50088. (b). ROC curves in GPL6480-OV, TCGA-OV, and GSE50088. (c). Map of risk score distribution in GPL6480-OV, TCGA-OV, and GSE50088.

We took GPL6480-OV (*n* = 558), TCGA-OV (*n* = 363), and GSE50088 (*n* = 109) as verification sets and found that the macrophage-associated prognostic model predicted OS in the three verification sets (Figure 6(a), *P* < 0.05). The AUC values of 3/5/7 years were greater than 0.5 (Figure 6(b)).  Figure 6(c) shows the distribution of risk scores in three cohorts. To verify the wide applicability and robustness of the model, we verified the model's predictive ability in another independent GSE26712 cohort of HGSOC. The model divided 185 patients into two groups (*P*=0.002, Supplementary Figure 4A), and the AUC values of 3/5/7 year were 0.624/0.595/0.564, respectively (Supplementary Figure 4B). Supplementary Figure 4C shows the risk distribution map of GSE26712 patients.

### 3.8. Construction of a Nomogram for Clinical Application

**Figure 7:**
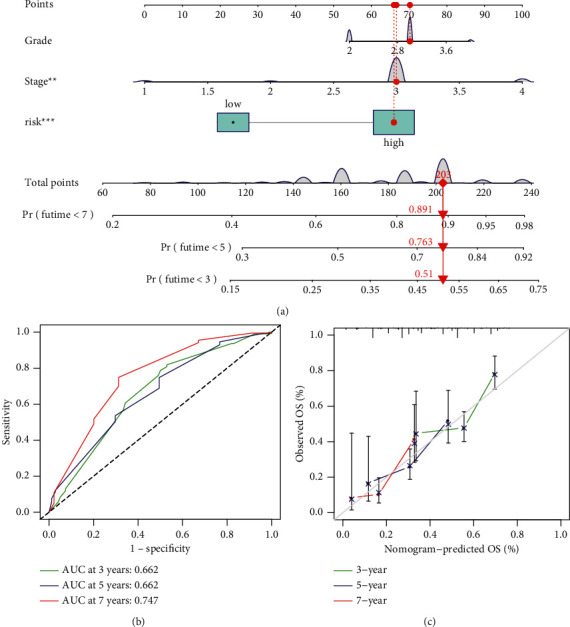
The nomogram. (a) Line chart. (b) ROC curve of the line chart. (c) Calibration curve of the line diagram. “∗” means *P* < 0.05, “^*∗∗*^” means *P* < 0.01, and “∗∗” means *P* < 0.001.

We built a nomogram diagram in the GPL570-OV cohort (Figure 7(a)). In this chart, there were three variables: clinical stage, pathological grade, and risk score used to calculate the 3-/5-/7-year survival rates. ROC and calibration curves were used to appraise the prediction ability of the line chart. The AUC values were all greater than 0.5, indicating that the nomogram had strong prognostic capacity (Figure 7(b)). The calibration curve shows that there was no obvious difference between the predicted value and the measured value (Figure 7(c)).

### 3.9. The Relationship between Macrophage-Related Model and Immunity

**Figure 8:**
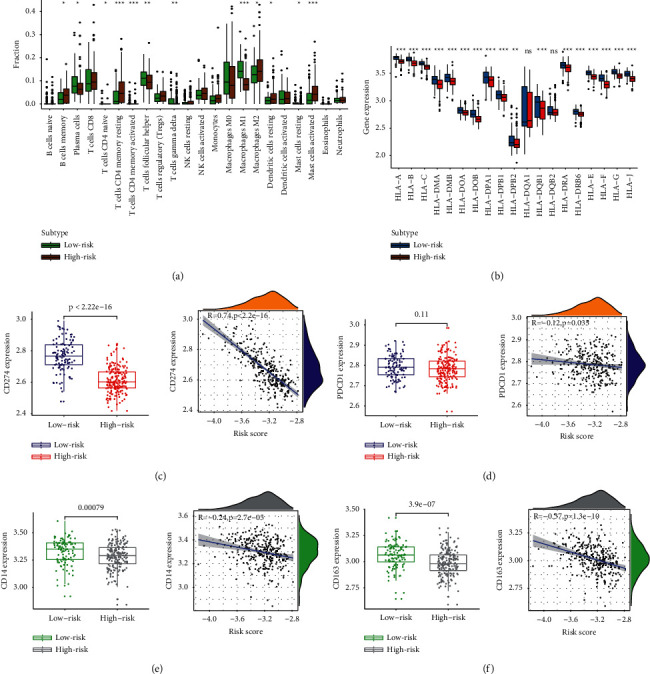
The relationship between risk score and immune factors. (a) The difference of 22 immune cells in high- and low-risk infiltration. (b) The difference of 19 HLA molecules in high- and low-risk infiltration. (c) The difference box diagram of CD274 in the low- and high-risk groups and the correlation scatter chart with the risk score. (d) PDCD1. (e) CD14. (f) CD163. “∗” means *P* < 0.05, “∗∗” means *P* < 0.01, and “∗∗” means *P* < 0.001.

We measured differences of infiltration in 22 leucocytes between high- and low-risk groups. We found that “CD4+ memory resting T cells,” “CD4+ memory activated T cells,” and “activated mast cells” showed higher infiltration levels in the high-risk group. In contrast, “M1 macrophages” showed higher infiltration levels in the low-risk group (Figure 8(a)). We also calculated the differences in the expression of 19 HLA (human lymphocyte antigen) molecules in high- and low-risk groups and found that these molecules were overexpressed in the high-risk group (Figure 8(b)). We then calculated the relationship between CD274/PDCD1 and risk scores, and CD274 showed higher expression in the low-risk group (Figure 8(c)). There was no statistical correlation between PDCD1 and risk score (Figure 8(d)). CD14 and CD163 are markers of macrophages. We explored their relationship with risk scores and found that the expression of these two genes was higher in the high-risk group and negatively correlated with the risk score (Figures 8(e)-8(f)).

### 3.10. Enrichment Analysis

**Figure 9:**
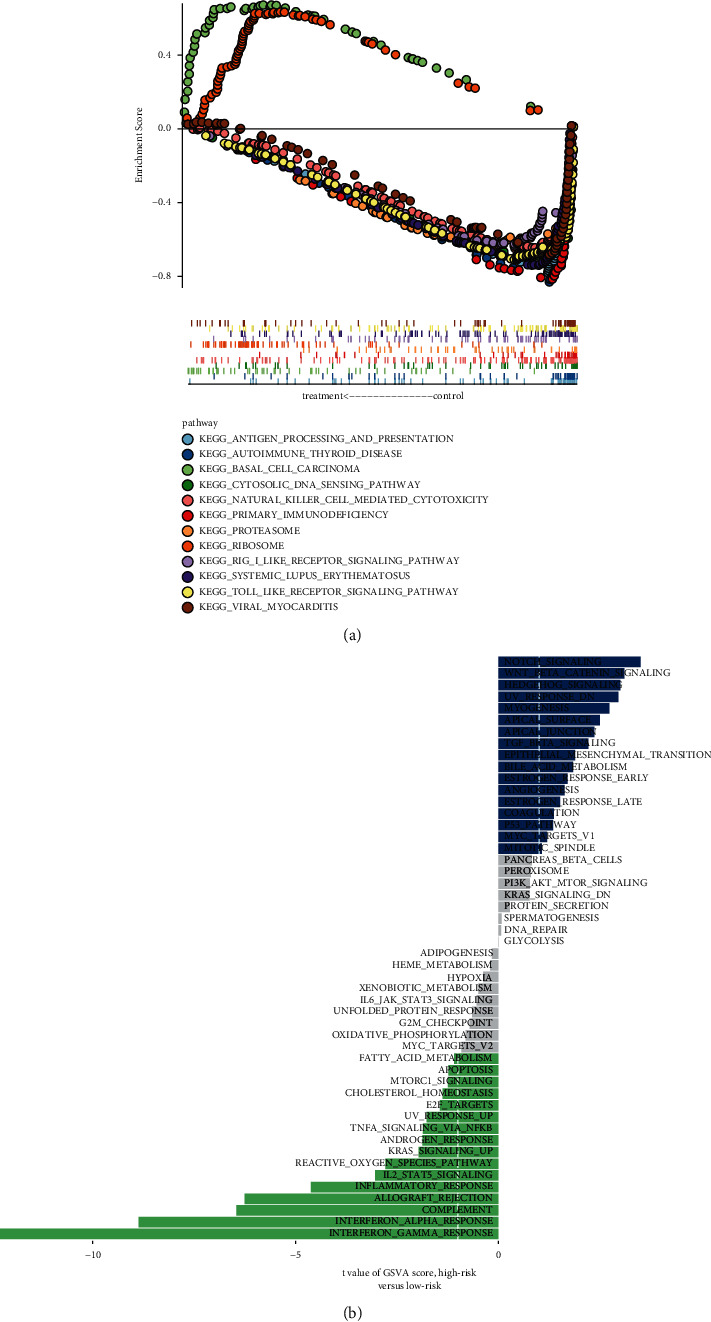
Enrichment analysis. (a) GSEA pathway enrichment analysis. (b) GSVA analysis.

We divided the GPL570-OV cohort into two groups for GSEA, and the gene set was “c2.cp.kegg.v7.0.symbols.” The pathways enriched in the high-risk group were “BASAL CELL CARCINOMA” and “RIBOSOME.” The low-risk group was enriched with “ANTIGEN PROCESSING AND PRESENTATION,” “AUTOIMMUNE THYROID DISEASE,” “CYTOSOLIC DNA SENSING PATHWAY,” “NATURAL KILLER CELL MEDIATED CYTOTOXICITY,” “PRIMARY IMMUNODEFICIENCY,” “PROTEASOME,” “RIG I LIKE RECEPTOR SIGNALING PATHWAY,” “SYSTEMIC LUPUS ERYTHEMATOSUS,” “TOLL LIKE RECEPTOR SIGNALING PATHWAY,” and “VIRAL MYOCARDITIS” (Figure 9(a)). Finally, we performed GSVA analysis of the high-risk and low-risk groups and found that “NOTCH SIGNALINGNOTCH SIGNALING” and “WNT BETA CATENIN SIGNALING” were enriched in the high-risk group, while “INTERFERON GAMMA RESPONSE” and “INTERFERON ALPHA RESPONSE” were enriched in the low-risk group (Figure 9(b)).

## 4. Discussion

HGSOC has the highest mortality among all gynecological cancers, and most cases show platinum-resistant recurrence [28]. Tumor microenvironment cannot be ignored in the pathogenesis and treatment of ovarian cancer [5]. In this study, we used the GPL570-OV cohort to explore the predictive ability of macrophages and found that both M1 and M2 macrophages predicted the OS of HGSOC. We used the WGCNA to identify the macrophage-related infiltration module and used the genes in the module to construct a prognostic model. The model can carry out risk stratification for 1566 HGSOC samples (*n* = 1566).

We constructed a macrophage-associated infiltration network. We calculated the degree of infiltration of M0/M1/M2 macrophages using CIBERSORT in the GPL570-OV cohort and measured the prognostic ability of these cells. We found that outcomes in patients with high levels of M1 macrophage infiltration were better, while those of patients with high M2 macrophage infiltration levels were worse. This is consistent with the fact that M1 macrophages play a role in tumor inhibition, while M2 macrophages help tumor immune escape. Then we used WGCNA to construct a gene coexpression network and found that black (M1) and green-yellow (M0/2) associated with macrophage infiltration. We selected the genes to construct the macrophage infiltration network, in which seven genes with high connectivity played a leading role in this network. RSAD2 is an antiviral protein that is significantly upregulated in M1 macrophages [29]. IFIT3 is a marker of M1 macrophage polarization and is highly upregulated in atherosclerosis and other inflammatory diseases [30]. In pneumonia, the microRNA network controls the replication of human macrophages through LGALS8 and MX1 [31]. IFIH1 contributes to the polarization of M1 macrophages in acute respiratory distress syndrome [32]. IFI44 is related to the migration and activation of macrophages [33]. ISG15 secreted by tumor cells increases tumor cell migration and immune escape by inducing M2 macrophage polarization [34]. In the case of ISG15 depletion, M1 macrophages show a robust proinflammatory cytokine expression pattern. These studies indicated that ovarian cancer cells may inhibit the polarization of M1 macrophages by inhibiting RSAD2, IFIT3, MX1, IFIH1, and IFI44 and induce the polarization of M2 macrophages by secreting ISG15, thereby promoting tumor progression. These genes are potential therapeutic targets for ovarian cancer.

We constructed a macrophage-related prognostic model of ovarian cancer. The model was made up of CD38, ACE2, BATF2, HLA-DOB, and WARS. Among these genes, WARS is highly expressed in the mRNA and protein levels of patients, as well as the mRNA level of cell lines, and the gene coefficient of WARS is the largest, indicating that WARS plays a core role in the model, while other genes play an auxiliary role. Studies showed that CD38 expression correlated with favorable outcomes by enhancing immune infiltration in the microenvironment of epithelial ovarian cancer [35]. ACE2, also known as ACEH and as angiotensin-converting enzyme 2, is a novel coronavirus cell surface receptor. Studies showed that ACE2 expression positively correlated with immunotherapy response and is a potential protective factor for ovarian cancer [36]. The ACE2/MAS1 axis is involved in the complex regulation of ovarian cancer function [37]. BATF2 has antitumor effects in many tumors: BATF2 combined with p53 to enhance protein stability in gastric cancer, thereby inhibiting ERK phosphorylation [38]. Upregulation of BATF2 inhibited human colorectal cancer cells' growth and epithelial-mesenchymal transformation [39]. BATF2 induced an antitumor effect in TAM by upregulating IL-12 expression [40]. The expression of HLA-DOB in multiple myeloma is significantly higher than that in normal plasma cells, suggesting that it is a potential target for immunotherapy [41]. Research shows that WARS compensates for the depletion of IFN-*γ*, thereby inhibiting tumor growth [42]. Single-cell data analysis of ovarian cancer showed that WARS was expressed in various immune cells, which may promote the infiltration of immune cells and inhibiting tumor. WARS may be an important tumor suppressor in OV, and further research on the mechanism is needed in the future.

Our macrophage-related model can carry out risk stratification of GPL570, GPL6480-OV, TCGA-OV, GSE50088, and GSE26712, with a sample size of 1566, indicating that the model is robust and widely applicable. In most previous studies, the scale of ovarian research was less than 1000 [43, 44], suggesting that these models are insufficiently accurate. The data from our study were derived from ten independent ovarian cancer cohorts worldwide, suggesting that our model is generalizable. These cohorts were strictly screened, leaving only HGSOC samples undergoing platinum therapy. Finally, we constructed a nomogram diagram in GPL570-OV which intuitively calculates the OS of HGSOC patients. The AUC values of 3/5/7 years were greater than 0.65, indicating that the nomogram diagram has a good predictive ability.

This model can help to explore the immune infiltration mechanism of HGSOC. CD4 memory T cells and activated mast cells showed higher infiltration levels in the high-risk group. In contrast, the degree of M1 macrophage infiltration increased significantly in low-risk group, indicating that CD4+ memory T cells and activated mast cells may be markers of poor outcome and may help tumor immune escape in the immune microenvironment. In contrast, M1 macrophage may be inhibited in high-risk patients. We then found that HLA molecules are overexpressed in high-risk groups, consistent with other studies on HLA molecules in ovarian cancer; HLA-G is a potential biomarker of advanced and complex ovarian cancer [45, 46]. HLA-G and HLA-E in ovarian cancer are potentially associated with the mechanism of disease progression [47]. Immune checkpoint inhibitors are new treatments for ovarian cancer. We found that the expression of CD247 increased in the low-risk group. CD247 is mainly expressed by tumor-infiltrating macrophages, not by malignant cells [48], which explains why CD247 is significantly overexpressed in low-risk patients.

Low-risk patients are enriched in immune-signaling pathways, suggesting that when immune-signaling pathways are active, patients are in a low-risk state. The results of GSEA analysis showed that the high-risk group was enriched in “BASAL_CELL_CARCINOMA” and “RIBOSOME,” suggesting that ovarian cancer and basal cell carcinoma have similar mechanisms. Recent studies showed that ribosomal ADP-ribosylation inhibits translation and maintains protein homeostasis in ovarian cancer; it is suggested that the ribosome may be an accomplice in the deterioration of ovarian cancer [49]. In the low-risk group of GSEA, many immune-signaling pathways were enriched, including NK cell killing, Toll-like receptor signals, antigen processing, and presentation. It is indicated that the immune system is highly activated in low-risk patients. At present, many studies have shown that the cancer vaccine triggers the immune response of ovarian cancer [5]. According to our research, we can try to use cancer vaccines in high-risk patients to activate the immune pathway. In GSVA analysis, NOTCH and WNT signals were enriched in the high-risk group, while interferon signal was activated in the low-risk group. PARP inhibitors have a specific therapeutic effect on ovarian cancer and show antitumor immunity, which occurs in a manner dependent on interferon gene stimuli and is enhanced by immune checkpoint blockade [50]. Therefore, PARP inhibitors combined with immune checkpoint blocking may be an effective treatment for ovarian cancer.

Although our model demonstrated excellent predictive power in 1566 patients with HGSOC, limitations remain. These ovarian cancer cohorts come from an online database and do not include our cohort, and samples should be expanded for verification before clinical application. The expression of some genes in the cohort is relatively low; therefore, highly sensitive detection techniques are needed in the future. The genes we screened did not further explore their mechanism in ovarian cancer, and further in vivo and in vitro experiments are needed in the future.

## 5. Conclusions

We constructed a relationship network related to macrophage infiltration that is helpful to explore the mechanisms of macrophages in HGSOC. We created a prognostic LASSO model based on macrophage-related genes. The model successfully predicted the OS of HGSOC in 1566 samples, which might be useful for assessing the condition and proper treatment of ovarian cancer.

## Figures and Tables

**Table 1:** The information of genes in the model.

Gene	Coefficient	Protein	Ensembl
CD38	−0.06314028104115	ADP-ribosyl cyclase/cyclic ADP-ribose hydrolase 1	ENSG00000004468
ACE2	−0.120878293790849	Angiotensin-converting enzyme 2	ENSG00000130234
BATF2	−0.100116694608065	Basic leucine zipper transcriptional factor ATF-like 2	ENSG00000168062
HLA-DOB	−0.0169011593879443	HLA class II histocompatibility antigen, DO beta chain	ENSG00000241106
WARS	−0.189999281219917	Tryptophan-tRNA ligase, cytoplasmic	ENSG00000140105

## Data Availability

The datasets used in the present study are available from the Cancer Genome Atlas database (http://cancergenome.nih.gov/) and GEO database (https://www.ncbi.nlm.nih.gov/geo/).

## Abbreviations

HGSOC: High-grade serous ovarian cancer
TCGA: The Cancer Genome Atlas
GEO: Gene Expression Omnibus
WGCNA: Weighted Gene Correlation Network Analysis
AUC: Area under the curve
ROC: Receiver operating characteristic
LASSO: Least absolute shrinkage and selection operator
coef: Regression coefficient
PFS: Progression-free survival
OS: Overall survival.

## Supplementary Materials

Supplementary Materials Supplementary Figure 1: differential expression of 5 genes in pan-cancer. (A) CD38. (B) ACE2. (C) BATF2. (D) HLA-DOB. (E) WARS. Supplementary Figure 2: the mRNA and protein expression level of the gene. (A) Differential expression of CD38 in 426 ovarian cancer tissues and 88 normal tissues. (B) ACE2. (C) BATF2. (D) HLA-DOB. (E) WARS. (F) CD38 pathological section of ovarian cancer tissue. (G) ACE2. (H) BATF2. (I) HLA-DOB. (J) WARS. (K) The expression level of 5 genes in ovarian cancer cell lines. Supplementary Figure 3: the study of genes at the single-cell level. (A) The enrichment correlation of ACE2, BATF2, and WARS with 14 pathways. (B) The distribution of CD38 in immune cells. (C) BATF2. (D) WARS. (E) HLA-DOB. Supplementary Figure 4: verify the robustness of the model in GSE26712. (A) Kaplan-Meier analysis of the model in GSE26712. (B) The ROC curve of the model in GSE26712. (C) The risk distribution map of the model in GSE26712.
