# Supplementary material for: Construction of a Macrophage Infiltration Regulatory Network and Related Prognostic Model of High-Grade Serous Ovarian Cancer
Source: J Oncol. 2021 Nov 24;2021:1331031. doi: 10.1155/2021/1331031 (PMC8635947; doi:10.1155/2021/1331031)

A

functional_states	ACE2	BATF2	WARS
Angiogenesis	0.4	0.089	0.174
Apoptosis	0.07	0.094	0.024
CellCycle	-0.091	-0.284	-0.127
Differentiation	0.262	0.154	0.227
DNAdamage	-0.399	-0.078	-0.073
DNArepair	-0.367	-0.17	-0.159
EMT	-0.103	0.078	0.029
Hypoxia	0.472	0.257	0.049
Inflammation	0.322	0.244	0.108
Invasion	-0.482	-0.373	-0.122
Metastasis	0.409	0.118	0.154
Proliferation	-0.241	0.085	-0.077
Quiescence	0.438	0.377	0.076
Stemness	0.087	-0.353	0.002

B

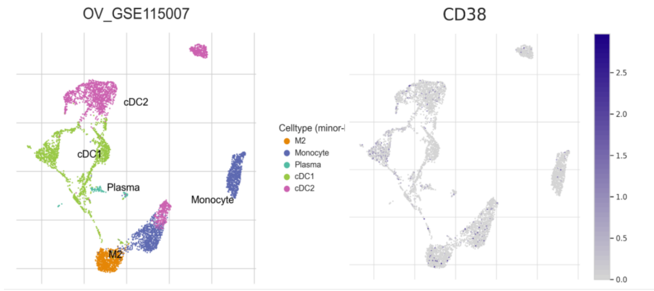

C

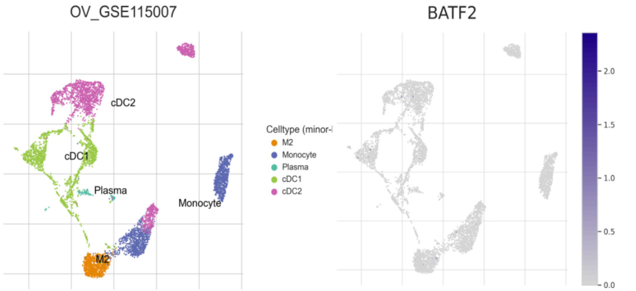

D

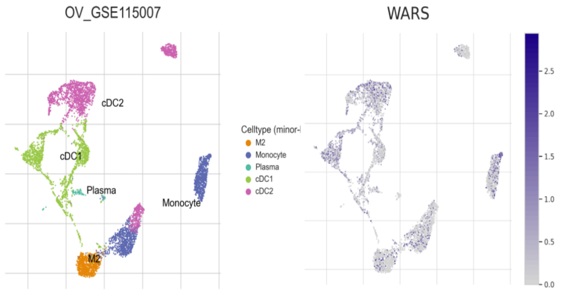

E

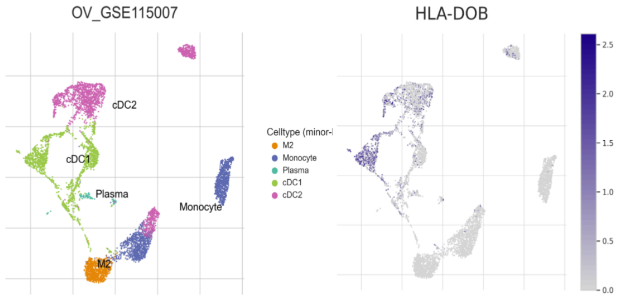

Supplement: Supplementary Materials — Supplementary Figure 1: differential expression of 5 genes in pan-cancer. (A) CD38. (B) ACE2. (C) BATF2. (D) HLA-DOB. (E) WARS. Supplementary Figure 2: the mRNA and protein expression level of the gene. (A) Differential expression of CD38 in 426 ovarian cancer tissues and 88 normal tissues. (B) ACE2. (C) BATF2. (D) HLA-DOB. (E) WARS. (F) CD38 pathological section of ovarian cancer tissue. (G) ACE2. (H) BATF2. (I) HLA-DOB. (J) WARS. (K) The expression level of 5 genes in ovarian cancer cell lines. Supplementary Figure 3: the study of genes at the single-cell level. (A) The enrichment correlation of ACE2, BATF2, and WARS with 14 pathways. (B) The distribution of CD38 in immune cells. (C) BATF2. (D) WARS. (E) HLA-DOB. Supplementary Figure 4: verify the robustness of the model in GSE26712. (A) Kaplan-Meier analysis of the model in GSE26712. (B) The ROC curve of the model in GSE26712. (C) The risk distribution map of the model in GSE26712. [file 1331031.f1.zip › 1331031.f1/Supplementary figure 3.pdf]
