# Supplementary figures and images for: Construction of a Macrophage Infiltration Regulatory Network and Related Prognostic Model of High-Grade Serous Ovarian Cancer
Source: J Oncol. 2021 Nov 24;2021:1331031. doi: 10.1155/2021/1331031 (PMC8635947; doi:10.1155/2021/1331031)

# A

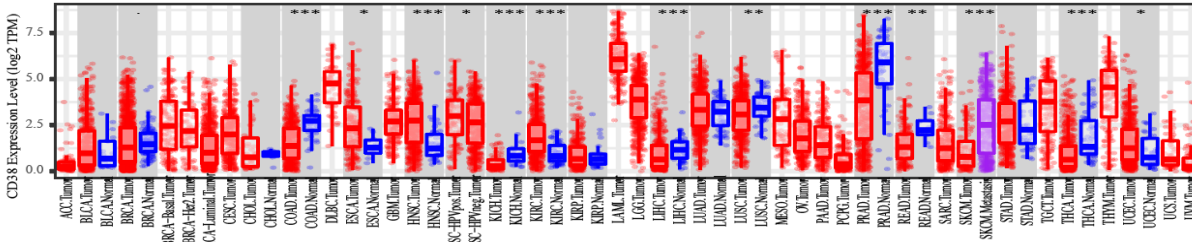

B

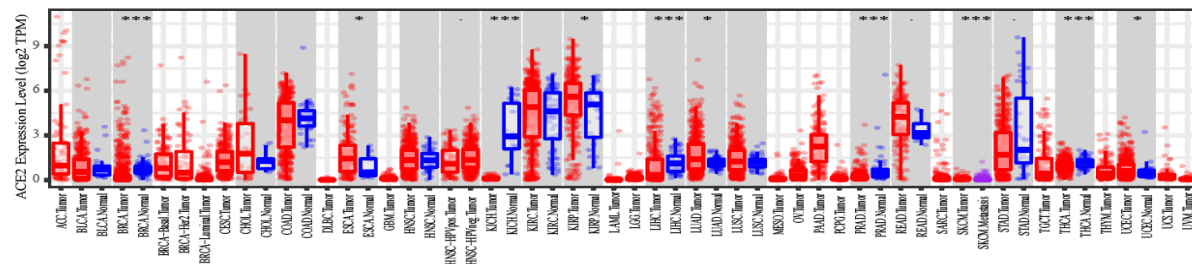

C

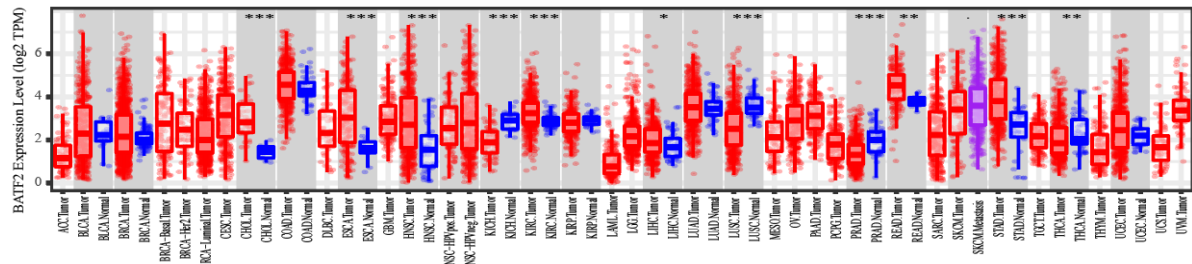

D

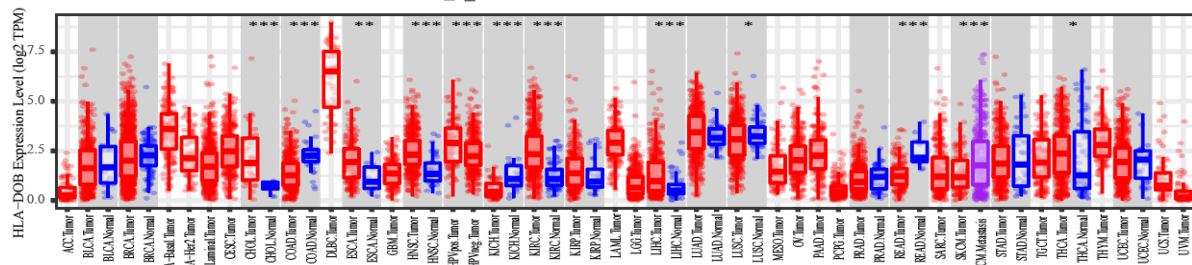

F

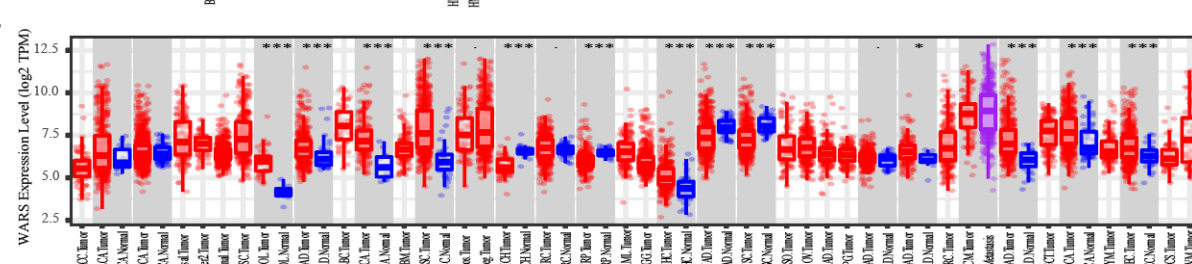

Supplement: Supplementary Materials — Supplementary Figure 1: differential expression of 5 genes in pan-cancer. (A) CD38. (B) ACE2. (C) BATF2. (D) HLA-DOB. (E) WARS. Supplementary Figure 2: the mRNA and protein expression level of the gene. (A) Differential expression of CD38 in 426 ovarian cancer tissues and 88 normal tissues. (B) ACE2. (C) BATF2. (D) HLA-DOB. (E) WARS. (F) CD38 pathological section of ovarian cancer tissue. (G) ACE2. (H) BATF2. (I) HLA-DOB. (J) WARS. (K) The expression level of 5 genes in ovarian cancer cell lines. Supplementary Figure 3: the study of genes at the single-cell level. (A) The enrichment correlation of ACE2, BATF2, and WARS with 14 pathways. (B) The distribution of CD38 in immune cells. (C) BATF2. (D) WARS. (E) HLA-DOB. Supplementary Figure 4: verify the robustness of the model in GSE26712. (A) Kaplan-Meier analysis of the model in GSE26712. (B) The ROC curve of the model in GSE26712. (C) The risk distribution map of the model in GSE26712. [file 1331031.f1.zip › 1331031.f1/Supplementary figure 1.pdf]

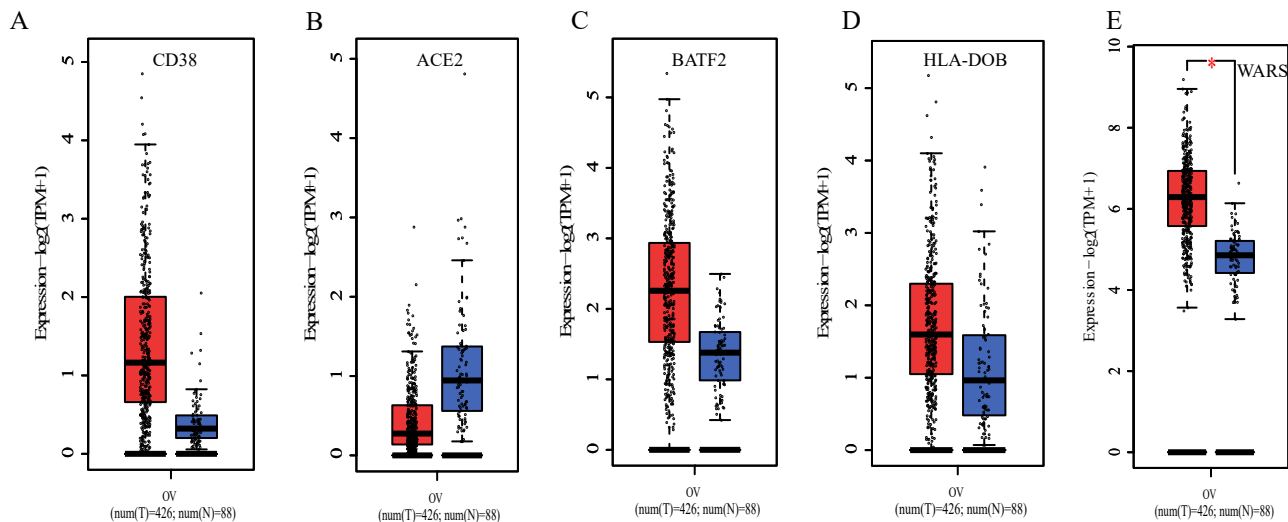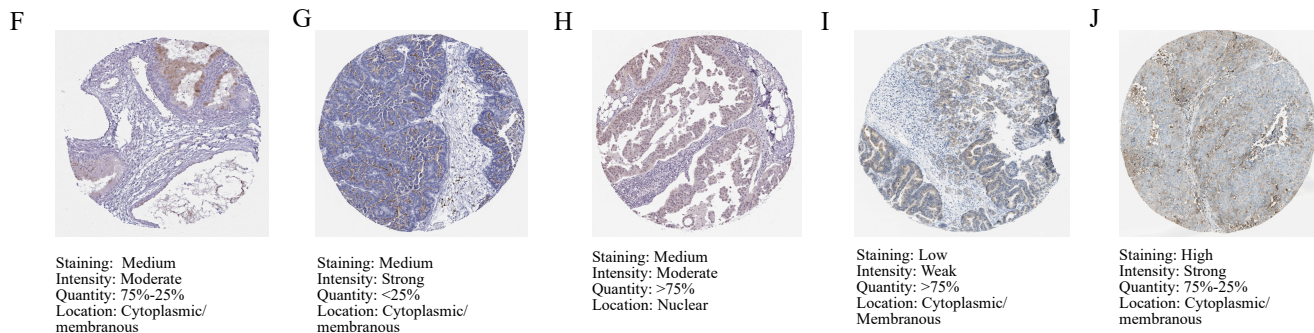

**K**

ACE2 BATF2 CD38 HLA-DOB WARS

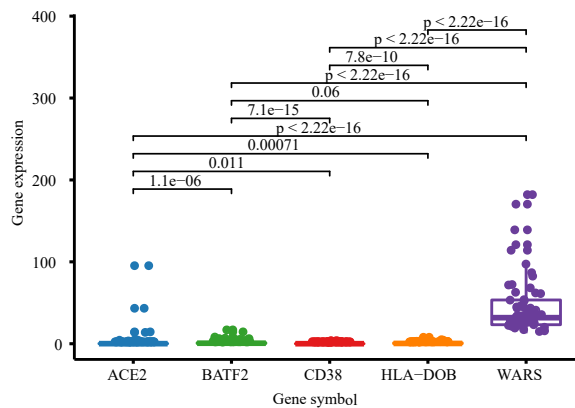

Supplement: Supplementary Materials — Supplementary Figure 1: differential expression of 5 genes in pan-cancer. (A) CD38. (B) ACE2. (C) BATF2. (D) HLA-DOB. (E) WARS. Supplementary Figure 2: the mRNA and protein expression level of the gene. (A) Differential expression of CD38 in 426 ovarian cancer tissues and 88 normal tissues. (B) ACE2. (C) BATF2. (D) HLA-DOB. (E) WARS. (F) CD38 pathological section of ovarian cancer tissue. (G) ACE2. (H) BATF2. (I) HLA-DOB. (J) WARS. (K) The expression level of 5 genes in ovarian cancer cell lines. Supplementary Figure 3: the study of genes at the single-cell level. (A) The enrichment correlation of ACE2, BATF2, and WARS with 14 pathways. (B) The distribution of CD38 in immune cells. (C) BATF2. (D) WARS. (E) HLA-DOB. Supplementary Figure 4: verify the robustness of the model in GSE26712. (A) Kaplan-Meier analysis of the model in GSE26712. (B) The ROC curve of the model in GSE26712. (C) The risk distribution map of the model in GSE26712. [file 1331031.f1.zip › 1331031.f1/Supplementary figure 2.pdf]

A

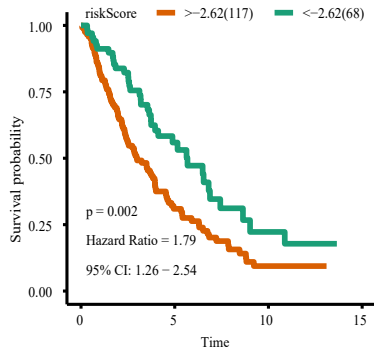

B

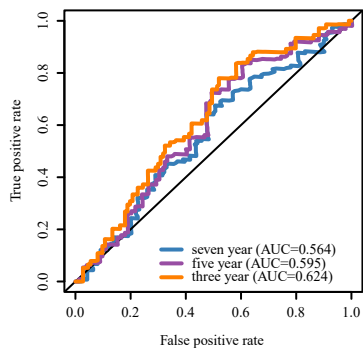

C

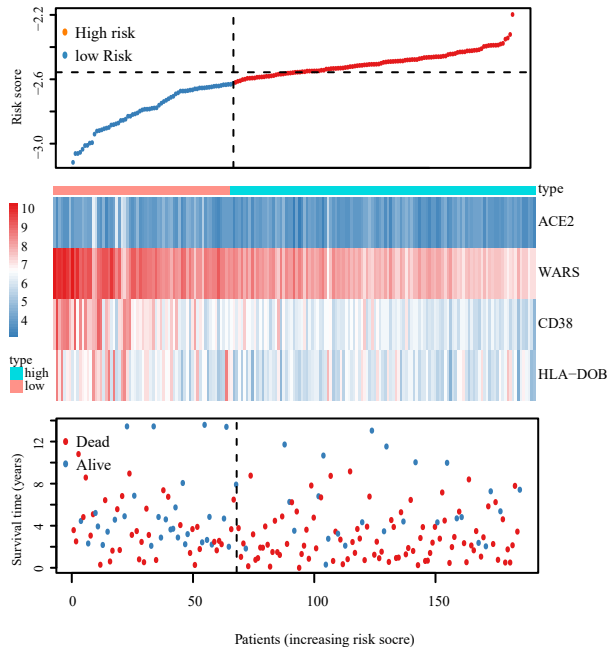

Supplement: Supplementary Materials — Supplementary Figure 1: differential expression of 5 genes in pan-cancer. (A) CD38. (B) ACE2. (C) BATF2. (D) HLA-DOB. (E) WARS. Supplementary Figure 2: the mRNA and protein expression level of the gene. (A) Differential expression of CD38 in 426 ovarian cancer tissues and 88 normal tissues. (B) ACE2. (C) BATF2. (D) HLA-DOB. (E) WARS. (F) CD38 pathological section of ovarian cancer tissue. (G) ACE2. (H) BATF2. (I) HLA-DOB. (J) WARS. (K) The expression level of 5 genes in ovarian cancer cell lines. Supplementary Figure 3: the study of genes at the single-cell level. (A) The enrichment correlation of ACE2, BATF2, and WARS with 14 pathways. (B) The distribution of CD38 in immune cells. (C) BATF2. (D) WARS. (E) HLA-DOB. Supplementary Figure 4: verify the robustness of the model in GSE26712. (A) Kaplan-Meier analysis of the model in GSE26712. (B) The ROC curve of the model in GSE26712. (C) The risk distribution map of the model in GSE26712. [file 1331031.f1.zip › 1331031.f1/Supplementary figure 4.pdf]
